# Influence of age, time of day, and environmental changes on vocalization patterns in broiler chickens

**DOI:** 10.1016/j.psj.2025.105298

**Published:** 2025-05-14

**Authors:** Patricia Soster de Carvalho, Tomasz Grzywalski, Kobe Buyse, Pieter Thomas, Camila Lopes Carvalho, Imad Khan, Bassem Khalfi, Frank Tuyttens, Maarten De Gussem, Paul Devos, Dick Botteldooren, Gunther Antonissen

**Affiliations:** a Department of Pathobiology, Pharmacology and Zoological Medicine, Faculty of Veterinary Medicine, Ghent University, 9090 Merelbeke-Melle, Belgium; b Poulpharm, Izegem, Belgium; c Department of Information Technology, Ghent University, Belgium; d Flanders Research Institute for Agriculture, Fisheries, and Food (ILVO), 9090 Merelbeke-Melle, Belgium; e Department of Veterinary and Biosciences, Faculty of Veterinary Medicine, Ghent University, 9090 Merelbeke-Melle, Belgium; f Vetworks, Aalter, Belgium

**Keywords:** Precision livestock farming, Poultry, Sound detection, Environmental enrichment, Heat stress

## Abstract

Sound analysis of animal vocalizations may provide valuable insights into their emotional state and behavioral responses to environmental stimuli. In broiler chickens, four primary vocalization types—distress calls (DC), short peeps (SP), warbles (W), and pleasure notes (PN)—are well-characterized through sound analysis. The objective of this study was to identify the normal vocalization patterns of broiler chickens throughout the on-farm stage, considering different times of the day (morning, midday, afternoon, and night), and to assess the effects of heat stress (HS) and multifunctional elevated platforms (MP) enrichment on these patterns. The trial included 1,680 male Ross 308 chickens, housed under either thermoneutral or HS conditions during the final two weeks, with half the pens enriched with MP. DC, SP, PN, and W were automatically monitored using custom-build broiler vocalization recognizer. When a vocalization did not fit one of the four categories, it was classified as another type of vocalization (OV). Time of day significantly influenced SP, DC, W, and PN. Throughout the starter, grower, and finisher phases, all treatments exhibited similar vocalization patterns across age and time of the day, highlighting the strong influence of daily periods on vocalizations. SP had the highest frequency in the evening and the lowest at night. DC peaked at midday, while W reached its highest frequency at night and was least frequent at midday. PN were most common at night but significantly lower during midday and evening. SP and DC were the most prevalent vocalizations, while PN occurred less frequently and were most prominent in broilers of 1 week old. W remained consistently low throughout the broilers’ lifespan. DC, SP, and OV increased with age, whereas PN declined, indicating a shift and more diverse vocal communication patterns as broilers mature. The presence of MP and HS did not influence vocalization patterns. These results demonstrate significant changes in vocalization patterns with age and time of the day throughout the on-farm cycle, while remaining relatively unaffected by the presence of MP or HS.

## Introduction

Farmers can often detect health and welfare problems in poultry houses by recognizing abnormal vocalization patterns (Bright, 2008; Herborn et al., 2020). However, the low minimal frequency required (European Union, 2007) and irregularity of farm inspections, along with the limited time spent in stables, introduce variability that makes consistent monitoring of flock welfare challenging. This challenge is compounded by the growing flock size in the poultry industry and a declining workforce responsible for their care, further complicating the effective oversight of large flocks, as reviewed by Norton et al. (2019).

Sound recording, combined with process engineering, offers a promising solution for developing efficient tools to monitor, control, and assess poultry health (Fontana et al., 2014). Microphones are capable of producing manageable amounts of data, making them a viable option for continuous real-time monitoring (Taffoni et al., 2018). In literature, four broiler chicken vocalization types - distress calls (DC), short peeps (SP), warbles (W), and pleasure notes (PN) - have been well-described through sound analysis (Marx et al., 2001). DC, which are marked by repetitive, high-intensity vocalizations, are closely linked to stress (Marx et al., 2001). SP are characterized by a descending frequency, with a waveform similar to DC, but with lower energy and a shorter duration (Marx et al., 2001), and are associated with periods of increased activity (Andrew, 1973). W are characterized by repeated, low and bow-shaped frequency contour that either rise or fall in frequency (Marx et al., 2001), suggesting a connection to drowsiness or a relaxed state (Guyomarc´h, 1966). PN are short-duration vocalizations characterized by an ascending frequency and a gentle upward swing in pitch, typically emitted with low energy (Marx. et al., 2001), potentially associated with positive welfare states (Guyomarc´h, 1966).

For sound analysis to serve as a reliable tool for real-time broiler monitoring, it is crucial to understand how vocalization patterns are influenced by age, diurnal rhythms, and how these patterns respond to environmental changes, such as heat stress (HS) challenge, and environmental enrichment as platforms. The thermoneutral zone is defined as the range of ambient temperatures where birds maintain their body temperature through sensible heat loss (IUPS, 2001). When ambient temperatures rise above this zone, broilers experience HS, as their ability to dissipate excess heat is overwhelmed (Alagawany et al., 2017). In response to HS, broilers exhibit behavioral changes, such as reduced feeding and movement, and increased panting, drinking, and resting with elevated wings (Mack et al., 2013).

Current debates define animal welfare as both the absence of negative states and the promotion of positive emotions. (Boissy et al., 2007; Muhammad et al., 2024; Vigors, 2019). Providing broilers with an enriched environment allows them to express natural behaviors (Newberry, 1995), which can help prevent negative emotions such as frustration and boredom while potentially promoting positive states (Silva et al., 2021). Roosting, resting, and sleeping are important behaviors for chickens, and they can benefit from having a suitable structure that supports these natural activities (Malchow and Schrader, 2021). Due to their heavier body conformation, broilers prefer elevated platforms rather than narrow perches that are commonly provided to laying hens, as they provide greater stability (Malchow and Schrader, 2021). Enrichment may also influence vocalization patterns, potentially serving as an indicator of welfare improvements (Tahamtani et al., 2016). To address key welfare challenges in broiler production such as HS, space limitations, and poor litter quality, we developed and tested multifunctional elevated platforms (MP) (Khalfi et al., 2024b).

Understanding the temporal evolution of vocalization patterns in broiler chickens as they age (from 1 to 42 days), across different times of the day (morning, midday, afternoon, and night), and under environmental changes, such as HS and platform presence, can provide deeper insights into the potential of automated recording and analyses of vocalizations for monitoring broiler welfare. The objectives of this trial were (i) to identify the normal vocalization patterns of broiler chickens throughout the on-farm stage, considering different times of the day (morning, midday, afternoon, and night), and (ii) to assess the effects of HS and MP on these patterns.

## Materials and methods

The experimental protocol for this study was approved by the Ethics Committee of the Flemish Research Institute for Agriculture, Fisheries, and Food (ILVO) in Merelbeke, Belgium, under authorization number 2022/414.

### Animals and treatments

The experiment consisted of three production rounds, each involving 560 male Ross 308 broiler chickens, for a total of 1,680 birds. In each round, the stable was divided into two compartments with independent automated climate control. Each compartment contained two pens of 36 m² (9 × 4 m). The pens had concrete floors covered with wood shavings (2.5 kg/m²). Our group developed and tested MP that integrate a cooling system for HS relief, dark shelters for resting, and manure collection trays to improve litter conditions (Khalfi et al., 2024a; Khalfi et al., 2024b). The MP were covered with wire mesh (mesh size 1.5 cm × 1.5 cm) and equipped with two plastic grid ramps (1.2 *m* × 0.28 m each; mesh size 19 mm × 40 mm) set at an 18° incline. A horizontal transition area connected the ramps to the platform (274 mm × 246 mm) (Fig. S1). In addition to providing a perching area, the platform created a sheltered, dark space in the area between the littered floor and the elevated platform (to mimic somewhat maternal protection, similar to an unheated dark brooder). This area was enclosed on all sides with 1 mm-thick black rubber, with fringed openings for bird entry and exit. To evaluate platform usage, a camera was installed near each platform in every pen. The number of chickens on each platform was counted every two hours between 7:00 AM and 9:00 PM, excluding those on the ramp and in the transition area.

**Fig. 1:**
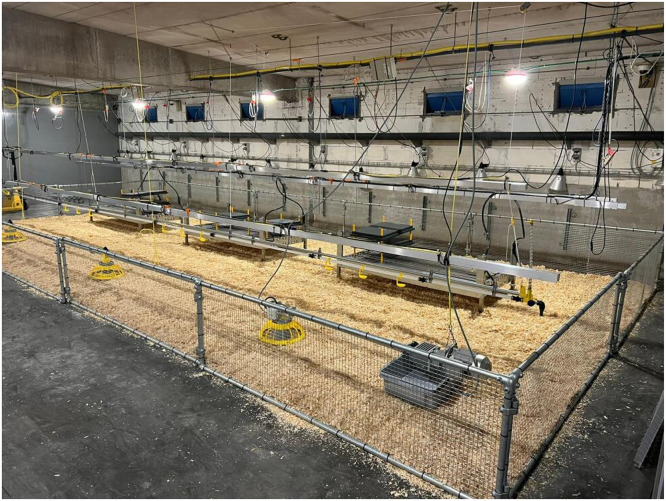
Layout of a broiler pen equipped with platforms, as used during the experimental trial.

In each compartment, one pen was equipped with three MP (Fig. 1), while the other pen served as control with no addition of multifunctional platforms (NMP). Each pen housed 140 broilers at a stocking density of 14 kg/m² or 3.8 birds/m². A lower density, than is common in conventional broiler production, was chosen in this trial to facilitate the identification of broiler vocalizations by reducing the overlap of multiple simultaneous calls. One-day-old chicks were randomly assigned to each pen. All birds received the same starter (days 0 to 9), grower (days 10 to 22), and finisher (days 23 to 41) feed. Feed and water were provided *ad libitum* and were formulated according to the nutritional recommendations of Ross 308. Birds were kept on a 23 light (L):1 dark (D) in the first week, and on an 18L6D light schedule between days 7 and 42. During the first week, the dark period was from 5:00 to 6:00 am. From the second week onwards, the dark periods were from 10:00 to 11:00 pm, 12:00 am to 4:00 am, and 5:00 to 6:00 am.

In the first week, the stable temperature was set at 32°C, after which it was gradually reduced by approximately 4°C per week until reaching 22°C at week 3, after which it was maintained through week 4. During the finisher phase, from days 29 to 33 and 36 to 40, HS was applied in one of the compartments, while the control compartment remained under thermoneutral conditions (TN). During the HS period, the temperature was set to increase from 22°C to 32°C, maintained from 9 AM to 3 PM, and then lowered back to 22°C. Measurements of humidity, temperature, and CO_2_ were recorded hourly in both compartments during the HS period. To evaluate HS, the temperature-humidity index (THI) was calculated using the formula from Buffington et al. (1981). Between rounds, the pens containing platforms and the compartment subjected to HS were rotated to ensure unbiased treatment effects across pens.

### Sound analysis

Two affordable sensor nodes, each equipped with a Knowles FG-23329 electret microphone (Van Renterghem et al., 2011), were placed 1.5 meters above the ground at the center of each pen to record 24/7 chicken vocalizations at a 48 kHz sampling rate. The microphones featured a frequency response of approximately 100 Hz to 10 kHz and an omnidirectional pickup pattern, making them suitable for capturing vocalizations across half of each pen area (covering 4.5 *m* × 4 m per microphone). The selected height balanced uniform recording coverage while minimizing background noise and reflections, thereby enhancing the signal-to-noise ratio. The choice of a 48 kHz sampling rate, supported by previous research (Huang et al., 2019; Liu et al., 2020; Lv et al., 2023), ensured the accurate capture of chicken vocalization frequencies. Recorded audio signals were processed using our custom-built broiler vocalization recognizer, as described in Soster et al. (2024), (2025)and Thomas et al., 2024.

To minimize cross-pen sound, the two pens located within the same compartment were separated by a distance of 3 meters to reduce the risk of recording vocalizations from birds in neighboring pens. Additionally, the two compartments were separated from the external environment by concrete walls, which helped block external sounds. A door between the compartments remained closed, further limiting sound transmission between pens.

The recognizer used in this study is based on a deep convolutional neural network (CNN) developed specifically for broiler vocalization detection (Soster et al., 2025). The architecture consists of eleven two-dimensional convolutional layers followed by a one-dimensional convolutional layer, comprising approximately 1.2 million trainable parameters. The model processes log-mel spectrogram representations of audio recordings and is designed to capture both the spectral and temporal features of broiler vocalizations. The final 1D convolutional layer produces seven outputs: five corresponding to vocalization classes, one for the absence of vocalization, and one to estimate the age of the broiler, which was introduced as an auxiliary training target to improve the model’s robustness by leveraging the age-dependent shift in vocalization pitch.

The model was initially pre-trained on a subset of 100,000 samples from the AudioSet database (Gemmeke et al., 2017), focusing on bird and fowl sounds, and later fine-tuned using a dedicated broiler vocalization database. This database was constructed from audio recordings of ten Ross 308 broilers, aged 1 to 36 days, housed in an acoustically isolated pen. Potential vocalizations were automatically preselected using the PANN model (Kong et al., 2020) and subsequently manually labeled into five categories: DC, SP, W, PN, and OV. The final dataset used for training and validation consisted of 2,559 labeled recordings, covering all weeks of the broiler lifecycle. At least 50 examples per vocalization type were included for each week wherever possible. To enhance performance under real-world conditions, the database was supplemented with recordings of background noise extracted from AudioSet, excluding animal sounds, allowing the model to learn to detect the absence of broiler vocalizations as a distinct class.

The recognizer was trained using noise-suppressed data, with a two-phase fine-tuning strategy and a multi-branch training approach designed to maximize knowledge transfer from pre-trained weights. The final model achieved an overall balanced accuracy of 91.1 % when classifying both vocalizations and background sounds, and a balanced accuracy of 89.4 % when distinguishing between specific broiler vocalization types (DC, SP, PN, W, OV). The model showed particularly high performance in detecting distress calls (97.1 % accuracy) and pleasure notes (98.5 % accuracy), demonstrating its suitability for reliable broiler vocalization monitoring.

Processing of the present trial recording with the vocalization recognizer included the following steps: (1) audio resampling to 8 kHz, (2) high-pass filtering using 5th-order Butterworth filter with a 500 Hz cutoff frequency, (3) non-stationary noise suppression using spectral gating (Sainburg et al., 2020), (4) conversion of the resulting audio signal into a log-mel spectrogram (64 mel filters, frequency range 50 Hz to 8 kHz, 100 spectrogram frames per second), and (5) processing the log-mel spectrogram with the trained neural network-based broiler vocalization recognizer. The model generated six probability scores (background, DC, PN, W, SP, and OV), which summed to 1.0.

The recordings were processed in 60-second segments, leveraging the fully convolutional nature of the neural network model to achieve high time-resolution predictions at one prediction every 240 ms. Next, the resulting probability rasters were analyzed to estimate the total duration of each vocalization type per minute. This was accomplished by applying a 0.7 probability threshold, experimentally determined to filter out uncertain detections, and counting the prediction frames where each vocalization type was identified. The total duration of each vocalization type per minute was estimated by multiplying the number of detected frames by the prediction frame duration (0.24 seconds). The reported frequency represents the proportion of the total observation time in which a particular vocalization was recorded. Finally, per-minute vocalization durations were aggregated hourly to minimize variation and enhance data stability and usability, with results expressed in seconds per minute (s/min).

### Statistical analysis

The statistical analyses were performed using R studio (version 2023.6.2.0). The pen was considered as the experimental unit for the entire trial with two microphones nested per pen. Pen and compartment were considered random effects. Data was analyzed using least-square linear regression with enrichment (MP vs NMP) and temperature in the finisher period (HS vs TN) as independent variables and the tested sound frequency as dependent variable. To evaluate the effects on THI values, ANOVA was conducted including treatment (HS vs. TN), round (1, 2, 3), and time of day (10AM, 12AM, 14PM) as fixed effects, along with their interactions. Post hoc comparisons were performed using Tukey’s HSD test.

Sound frequencies were averaged based on categorical time periods during the day: night (10 PM to 4 AM), morning (4 AM to 10 AM), midday (10 AM to 4 PM), and evening (4 PM to 10 PM). Notably, the night period encompassed the majority of the dark phase. The interaction effect between time periods and treatments (HS and MP) was removed from the final model when not significant. If significant effects were found, post-hoc pairwise comparison tests with Tukey correction were performed to assess differences between treatments. The data was checked for outliers and checked for a normal distribution of the residuals. Differences were considered statistically significant at *p* < 0.05. Mean diurnal patterns per hour and evolution throughout the rearing period of each sound type were assessed visually. Spline regression with standard error was used to visualize the evolution during the day.

## Results

### Time of the day: starter, grower and finisher phase

Diurnal and night patterns in vocalizations were seen for the continuous monitoring of the six weeks of the broilers’ lives. SP showed a clear diurnal rhythm, with a sharp increase in the morning, and peaks during the day before gradually declining in the evening and night. DC also showed a clear diurnal rhythm with peaks at midday. PN peaked in the morning and evening, while W reached its highest frequency at night and was least frequent at midday.

The time of day effect on SP, DC, W, and PN was significant (*p* = 0.001) in the starter period (Table 1). SP were most frequent in the evening and least at night in both treatments. In NMP, it ranged from 17.4 to 29.7 s/min, and in MP, from 18.6 to 30.8 s/min. DC peaked at midday in both treatments (4.54 s/min in NMP and 4.43 s/min in MP) and dropped to 1.11 s/min at night. W showed the lowest overall frequency but was highest at night (0.154 s/min in NMP and 0.161 s/min in MP) and lowest at midday (0.063 s/min in NMP and 0.015 s/min in MP). PN occurred more at night and in the morning, with peaks of 6.77 s/min (NMP) and 7.13 s/min (MP) at night, and the lowest values in the evening. OV remained stable across all times and treatments (*p* = 0.759), ranging from 0.379 to 0.527 s/min.

**Table 1:** Frequency (s/min) of broiler chicken vocalizations in the starter phase (from 0 to 9 days) across treatments and time of day categorized by time periods during the day: night (10 PM to 4 AM), morning (4 AM to 10 AM), midday (10 AM to 4 PM), and evening (4 PM to 10 PM). The vocalizations include distress calls (DC), pleasure notes (PN), short peeps (SP), warbles (W), and other types of vocalizations (OV).

Treatment	Time of day	Sound type – Starter Phase				
		SP (s/min)	DC (s/min)	W (s/min)	PN (s/min)	OV (s/min)
NMP	morning	17.7 ^c^	2.51 ^b^	0.112 ^b^	6.24 ^a^	0.455
	midday	27.3 ^b^	4.54 ^a^	0.063 ^c^	3.33 ^b^	0.382
	evening	29.7 ^a^	2.74 ^b^	0.067 ^c^	2.48 ^b^	0.434
	night	17.4 ^c^	1.11 ^c^	0.154 ^a^	6.77 ^a^	0.527
MP	morning	18.9 ^c^	2.39 ^b^	0.119 ^b^	6.36 ^a^	0.442
	midday	28.4 ^b^	4.43 ^a^	0.015 ^c^	3.41 ^b^	0.379
	evening	30.8 ^a^	2.63 ^b^	0.074 ^c^	2.60 ^b^	0.433
	night	18.6 ^c^	1.11 ^c^	0.161 ^a^	7.13 ^a^	0.458
SEM		0.931	0.163	0.018	0.316	0.061
*P-values*	treatment (B)	0.217	0.423	0.541	0.782	0.782
	time (T)	0.001	0.001	0.001	0.001	0.759
	B*T	0.873	0.249	0.978	0.923	0.997

Superscripts a-c within a column indicate significant differences (*p* < 0.05). No pairwise differences with OV were included in the analysis.

MP: multifunctional platforms. NMP: no addition of multifunctional platforms.

Time of the day significantly affected SP, DC, W, and PN during the grower phase (*p* = 0.001; Table 2). SP peaked in the evening and was lowest at night in both treatments, ranging from 9.28 to 23.29 s/min in NMP and 9.07 to 25.63 s/min in MP. DC were highest at midday (10.37 s/min in NMP, 10.32 s/min in MP) and lowest at night (2.45 and 2.20 s/min, respectively). W had the lowest overall frequency but was most frequent at night and least at midday, ranging from 0.014 to 0.047 s/min in NMP and 0.017–0.050 s/min in MP. PN also peaked at night (1.275 s/min in NMP, 1.290 s/min in MP) and was least common at midday. OV showed no significant variation across time (*p* = 0.797) or treatment (*p* = 0.200), remaining stable between 0.466 and 0.606 s/min.

**Table 2:** Frequency (s/min) of broiler chicken vocalizations in the grower phase (from 10 to 22 days) across treatments, categorized by time periods during the day: night (10 PM to 4 AM), morning (4 AM to 10 AM), midday (10 AM to 4 PM), and evening (4 PM to 10 PM). The vocalizations include distress calls (DC), pleasure notes (PN), short peeps (SP), warbles (W), and other types of vocalizations (OV).

Treatment	Time of day	Sound type – Grower Phase				
		SP (s/min)	DC (s/min)	W (s/min)	PN (s/min)	OV (s/min)
NMP-TN	morning	18.95 ^ab^	6.12 ^b^	0.027 ^b^	0.577 ^b^	0.502
	midday	23.08 ^ab^	10.37 ^a^	0.014 ^c^	0.149 ^c^	0.466
	evening	23.29 ^ab^	9.57 ^a^	0.016 ^bc^	0.150 ^c^	0.493
	night	9.28 ^cd^	2.45 ^c^	0.047 ^a^	1.275 ^a^	0.490
						
MP-TN	morning	20.22 ^ab^	6.04 ^b^	0.031 ^b^	0.593 ^b^	0.549
	midday	25.10 ^ab^	10.32 ^a^	0.017 ^c^	0.164 ^c^	0.540
	evening	25.63 ^a^	9.65 ^a^	0.018 ^c^	0.165 ^c^	0.606
	night	9.07 ^d^	2.20 ^c^	0.050 ^a^	1.290 ^a^	0.504
SEM		1.56	0.520	0.004	0.084	0.050
*P-values*	treatment (B)	0.969	0.942	0.194	0.391	0.200
	time (T)	0.001	0.001	0.001	0.001	0.797
	B*T	0.905	0.957	0.989	0.105	0.870

Superscripts a-c within a column indicate significant differences (*p* < 0.05). No pairwise differences with OV were included in the analysis.

MP: multifunctional platforms. NMP: no addition of multifunctional platforms.

In the finisher phase, time of day significantly influenced broiler vocalizations (Table 3). SP were highest in the evening and lowest at night across treatments, ranging from 5.11 to 13.04 s/min in NMP-TN and 5.28–13.31 s/min in MP-HS. DC peaked at midday and evening (*p* = 0.006), with the highest frequency in MP-HS at midday (17.33 s/min) and the lowest in MP-TN at night (4.93 s/min). W remained the least frequent vocalization but followed a consistent pattern, peaking at night and dropping at midday, with the highest value in MP-TN at night (0.027 s/min). PN were more frequent at night, reaching 0.41 s/min in MP-HS, and were significantly lower at midday and evening. OV remained relatively stable throughout the day, with slightly higher evening values, peaking at 3.37 s/min in MP-TN. No significant differences were found in vocalization patterns between HS and TN conditions or between MP and NMP treatments (*p* > 0.05), indicating limited effects of these factors on vocal behavior.

**Table 3:** Frequency (s/min) of broiler chicken vocalizations in the finisher phase (from 22 to 41 days) across treatments, categorized by time periods during the day: night (10 PM to 4 AM), morning (4 AM to 10 AM), midday (10 AM to 4 PM), and evening (4 PM to 10 PM). The vocalizations include distress calls (DC), pleasure notes (PN), short peeps (SP), warbles (W), and other types of vocalizations (OV).

Treatment	Time of day	Sound type – Finisher Phase				
		SP (s/min)	DC (s/min)	W (s/min)	PN (s/min)	OV (s/min)
NMP-TN	morning	10.35 ^abc^	11.96 ^ab^	0.013 ^ab^	0.169 ^b^	2.54 ^ab^
	midday	12.16 ^abc^	16.72 ^a^	0.007 ^b^	0.051 ^c^	2.81
	evening	13.04 ^ab^	15.95 ^a^	0.008 ^b^	0.063 ^c^	3.07 ^a^
	night	5.11 ^d^	5.5 ^b^	0.019 ^a^	0.394 ^a^	1.79 ^b^
NMP-HS	morning	9.21^c^	11.44 ^ab^	0.014 ^ab^	0.146 ^b^	2.52 ^ab^
	midday	9.85^bc^	15.76 ^a^	0.006 ^c^	0.027 ^c^	2.63 ^ab^
	evening	11.31 ^abc^	15.94 ^a^	0.008 ^bc^	0.047 ^c^	3.06 ^a^
	night	4.85 ^d^	5.41 ^b^	0.021 ^a^	0.341 ^a^	1.95 ^b^
MP-TN	morning	10.52 ^abc^	11.7 ^ab^	0.017 ^b^	0.164 ^b^	2.16 ^ab^
	midday	11.09 ^abc^	15.87 ^a^	0.008 ^c^	0.057 ^c^	2.97 ^ab^
	evening	12.68 ^ab^	16.22 ^a^	0.01 ^bc^	0.057 ^c^	3.37 ^a^
	night	5.46 ^d^	4.93 ^b^	0.027 ^a^	0.368 ^a^	2.1 ^b^
MP-HS	morning	11.05 ^abc^	12.69 ^ab^	0.015 ^b^	0.183 ^b^	2.5 ^ab^
	midday	12.7 ^abc^	17.33 ^a^	0.007 ^b^	0.044 ^c^	2.85 ^ab^
	evening	13.31 ^a^	16.03 ^a^	0.009 ^b^	0.057 ^c^	3.2 ^a^
	night	5.28 ^d^	5.42 ^b^	0.024 ^a^	0.41 ^a^	1.99 ^b^
SEM		0.643	0.937	0.001	0.063	0.180
*P-values*	treatment (B)	0.083	0.006	0.361	0.167	0.631
	time (T)	0.001	0.001	0.001	0.001	0.004
	B*T	0.949	0.988	0.998	0.580	0.964

Superscripts a-c within a column indicate significant differences (*p* < 0.05). No pairwise differences with OV were included in the analysis.

MP: multifunctional platforms. NMP: no addition of multifunctional platforms.

### Vocalizations patterns with age

**Fig. 2:**
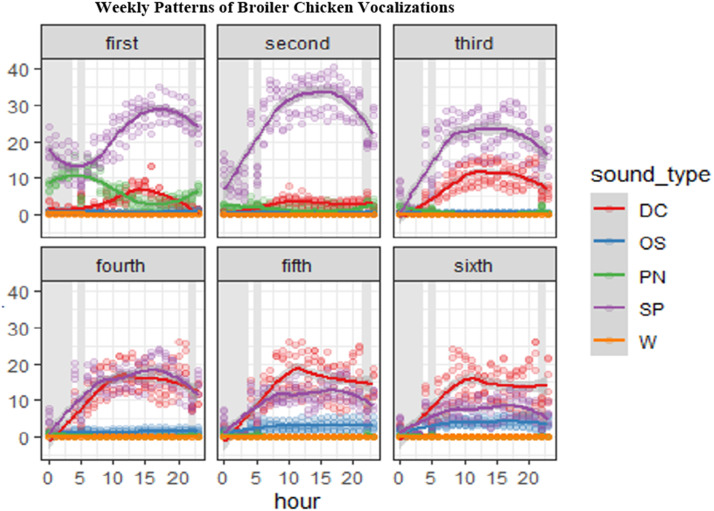
Weekly frequency (s/min) of continuous vocalizations in broiler chickens, including distress calls (DC), pleasure notes (PN), short peeps (SP), warbles (W), and other types of vocalizations (OV) from 1 to 42 days of age. The grey color represents the dark period.

The results demonstrate a clear transition in vocalization patterns with age (Fig. 2). Young broilers primarily emitted SP and PN, while older birds increasingly produced DC. During the first three weeks, vocal activity was dominated by SP. PN were also present early on but declined noticeably after the third week. In contrast, DC were infrequent in the first two weeks, began increasing in the third week, and became more prominent from the fourth week onward. By the fifth and sixth weeks, DC had surpassed SP in frequency, particularly during afternoon hours. W and OS remained consistently low across all weeks. However, OS presented a slight increases in older birds.

### Heat stress period

No significant differences (*p* > 0.05) in vocalization patterns were observed in the finisher phase, when broilers were subjected to HS. Throughout the three experimental rounds, distinct differences in THI were observed between the compartments, particularly between the one subjected to HS and the TN condition (Table S1). In the HS compartment, THI values ranged from 23.0 to 26.1 during round 1 (winter), increased up to 27.0 in round 2 (spring), and peaked up to 28.6 in round 3 (summer). In contrast, the TN compartment maintained lower THI values across all rounds (round 1: 17.8–20.0, round 2: 18.4–20.6, and round 3: 19.3–25.2). The mean THI was significantly higher in the HS condition compared to the TN condition (*p* < 0.001). Regarding the rounds, Round 3 presented significantly higher THI values than Round 1 (*p* < 0.001) and Round 2 (*p* = 0.0022), while the difference between Round 1 and Round 2 was not statistically significant (*p* = 0.1151). No significant differences were found across the time points of 10AM, 12AM, and 14PM (all *p* > 0.84).

### Multifunctional platforms

Across all rounds, bird use of the platforms increased steeply during the first two weeks of life, peaking at around seven birds per platform between days 15 and 20 (Fig. S2). After this period, the number of birds remained relatively stable toward the end of the production cycle. However, no significant differences (*p* > 0.05) in vocalization patterns were observed across any evaluated phase when broilers were provided with MP.

## Discussion

The present study revealed a clear pattern of vocalizations throughout the broilers’ lifespan. Most vocalizations were classified as SP, DC, PN, or W. OV remained low but showed a slight increase after the third week of age. SP and PN declined after the first week, DC decreased after week four, and W remained consistently low. Time of day significantly affected SP, DC, PN, and W, but not OV. Overall, SP and DC were the most frequent vocalizations, PN appeared less often with occasional peaks, and W remained rare. These trends suggest an age-related shift in vocal expression, with increased DC and SP and declining PN as broilers mature. However, no significant differences related to HS or MP could be demonstrated in this study.

DC and SP emerged as the most frequent vocalizations throughout the study, with SP dominating during the first two weeks. This call is associated with active behaviors such as exploration, foraging, and social interaction (Andrew, 1973). The subsequent decline in SP may reflect reduced activity as broilers gain weight, which is known to limit mobility (Bizeray et al., 2000). Early in life, birds typically exhibit low gait scores (1–2), indicating better locomotion and higher activity levels (Pena Fernández et al., 2018). By the finisher phase, increased body weight and declining litter quality (Riber and Wurtz, 2024) may further reduce movement and SP frequency. However, further research is needed to confirm the function of SP, which could offer useful insights into activity levels and positive behaviors such as dustbathing, wing flapping, and exploration.

DC in broiler chickens are widely regarded as urgent vocal signals associated with negative emotional states or physical discomfort (De Moura et al., 2008; Pereira et al., 2023). However, in the present study DC emerged as the second most frequent vocalization, and their incidence increased from the third week onward despite low stocking density and otherwise favorable husbandry. This pattern suggests that DC may not be elicited exclusively by stressors. Instead, they could reflect a state of heightened arousal that is not necessarily negative. For comparison, high corticosterone levels are known to indicate both negative stress and positive excitement, depending on the context (Sapolsky, 2000). Similarly, DC might capture a broader range of emotional states. As highlighted by Miller et al. (2022), the structure and function of avian DC are complex and may reflect a broader range of emotional states. Therefore, DC may serve as a more nuanced indicator of broiler condition rather than a direct marker of poor welfare. The concurrent rise in OV with age may further reflect vocal maturation and an expanding repertoire.

In addition to this shift in vocal expression with age, a distinct pattern was observed in the daily timing of vocalizations. The higher frequency of vocalizations during the day suggests a clear diurnal rhythm in broiler vocal behavior, aligned with natural activity-rest cycles (Deep et al., 2010). Vocalizations decreased at night, reflecting reduced activity during the resting period (Sibanda et al., 2019), a pattern also observed by Du et al. (2018), who reported over 53 vocalizations per day in healthy layers, but fewer than one at night. In the present study, SP and DC peaked during the day, while PN and W were more frequent at night. This may indicate a link between PN and W and resting periods, or reflect the reduced presence of louder calls (SP and DC) at night, allowing quieter vocalizations to be more easily detected (Soster et al., 2024).

Beyond circadian influences, environmental conditions were also evaluated as potential modulators of vocal behavior. Despite exposure to HS conditions, broiler vocalization patterns remained unchanged in this study. Although a consistent target temperature was applied, daily and seasonal variations led to fluctuating THI values and inconsistent HS exposure. While previous studies have associated high temperatures with increased vocal activity in chickens (Du et al., 2020; Gandra et al., 2020; Mortola, 2019), they often lack detailed descriptions of vocal types and THI thresholds. Traditionally, THI values above 27.8 indicate moderate to severe HS (Marai et al., 2001), with negative effects on broiler performance reported beyond 27 (Purswell et al., 2012; Vale et al., 2010). However, modern broilers, selected for rapid growth and high metabolic rates, are likely more sensitive to heat due to increased internal heat production and reduced capacity for heat dissipation (Nawaz et al., 2021; Teyssier et al., 2022). Recent studies have reported performance declines at THI values around 21 (Kim et al., 2024; Purswell et al., 2012), suggesting that HS may have occurred even during Round 1. Nonetheless, uncertainty about the extent of HS induction, particularly in Rounds 1 and 2, may explain the absence of observable changes in vocal behavior.

Environmental enrichment was also considered, though its impact on vocal behavior likely depends on the level of interaction and measurement context. In our study, no significant differences in vocalization patterns were found between pens with and without MP. From the second week onward, MP use averaged about seven birds per platform, or 21 per pen (only around 15 % of the flock). This limited engagement may have been insufficient to influence vocal behavior. However, previous studies have shown that enrichment can impact vocalizations: Golfidis et al. (2024) linked vocal changes to emotional stimuli via interactive feeders, and Meyer et al. (2024) reported reduced vocalizations under laser enrichment. Additionally, our data were not collected during acute stress events like fear tests or isolation, which typically elicit stronger vocal responses (Zimmerman et al., 2011). These factors may also explain the absence of enrichment-related effects in our findings.

This study demonstrates the potential of using a custom-built broiler vocalization recognizer to automatically monitor the four main broiler chickens vocalizations 24/7 in real-time, both on-farm and in future research. By detecting and analyzing the most frequent vocalizations throughout the production cycle, this approach can offer valuable insights into the birds' internal states and behavioral patterns. It may also provide useful information on diurnal and nocturnal rhythms, as well as age-related changes in vocal behavior. DC, easily recognized by their high frequency and often linked to stress, increased from the third week onward, even under favorable conditions. Alongside a rise in OV, this may reflect a shift in vocal behavior with age, suggesting these calls signal more than just distress. SP, which follow a strong diurnal pattern and are most frequent during the first two weeks, may reflect activity levels. PN, primarily detected at night during the first week (when birds are presumably in a relaxed state) could be linked to positive emotional states. W, though possibly associated with positive welfare, are less frequent and often masked by louder vocalizations, making them more difficult to assess.

This study offers valuable insights into broiler vocalization patterns across the production cycle. However, limitations include the presence of OV with unclear meanings, though their increase mid-cycle suggests a potential role in communication. The experiment was conducted under otherwise favorable conditions, apart from the induced HS, which may not fully reflect the challenges of commercial production systems. Future studies should assess vocalizations in commercial settings and explore how they respond to significant stressors, as well as further clarify the significance of each vocalization type.

## Conclusions

The developed method proved to be sensitive and effective in detecting the vocalizations in broiler chickens across different age stages, showing a strong diurnal rhythm of vocalizations. SP were the most frequent vocalization but declined with age, while DC increased from the third week, coinciding with rapid growth and possible welfare challenges. PN was more common early in life and occurred mainly at night, possibly indicating a positive emotional state. Its decline, along with rising DC and OV, suggests a shift in vocal behavior and possibly a broader vocal repertoire as broilers mature. W remained consistently low, likely masked by louder calls. No effect of platform use or HS was observed on vocalizations.

This study demonstrates the potential of using a custom-built broiler vocalization recognizer to automatically monitor broiler chickens 24/7, both on-farm and in future research. By detecting and analyzing the most frequent vocalizations throughout the production cycle, this approach can offer valuable insights into the birds' internal states and behavioral patterns. It may also provide useful information on diurnal and nocturnal rhythms, as well as age-related changes in vocal behavior. Future research should assess vocalization patterns in commercial settings and explore how they change in response to various stressors. Also, further investigation into the significance of OV could enhance the understand chickens' communication.

## Declaration of competing interest

The authors declare no interests/personal relationships which may be considered as potential competing interests.

## Appendix Supplementary materials

Image, application 1

Image, application 2

Image, application 3
