# Supplementary material for: Influence of age, time of day, and environmental changes on vocalization patterns in broiler chickens
Source: Poult Sci. 2025 May 14;104(8):105298. doi: 10.1016/j.psj.2025.105298 (PMC12173062; doi:10.1016/j.psj.2025.105298)
Supplement: Supplementary file 1 [file mmc1.docx]

**SUPPLEMENTARU MATERIAL**

Supplementary figure 1: Design of the platform used in the trial.

Supplementary figure 2: Average number of chickens observed on the platforms throughout the production cycle (days 1 to 42) across the three experimental rounds: no multifunctional platform under thermoneutral conditions (NMP-TN), no multifunctional platform under heat stress (NMP-HS), multifunctional platform under thermoneutral conditions (MP-TN), and multifunctional platform under heat stress (MP-HS). Data represent means with shaded areas indicating confidence intervals. Number of birds were counted every two hours from 7:00 AM to 9:00 PM using overhead cameras.

Supplementary table 1: Mean temperature-humidity index (THI) values recorded over 10 days under heat stress and thermoneutral conditions across three experimental rounds and three time points per day (10:00 AM, 12:00 PM, and 2:00 PM), corresponding to the period of heat stress application.

|  | Heat Stress | | | Thermoneutral | | |
| --- | --- | --- | --- | --- | --- | --- |
| Time | Round 1 | Round 2 | Round 3 | Round 1 | Round 2 | Round 3 |
| 10AM | 24,38 | 25,97 | 26,94 | 18,64 | 19,62 | 21,67 |
| 12AM | 24,66 | 25,95 | 27,4 | 18,81 | 19,5 | 22,28 |
| 14PM | 24,67 | 26,25 | 27,39 | 18,87 | 19,47 | 22,58 |
| Mean | 24,61^b^ | 25,84 ^b^ | 27,25^a^ | 18,86^d^ | 19,46 ^d^ | 22,03^c^ |

Superscript letters (a, b, c, d) indicate statistically significant differences between groups (p < 0.05).
