# Supplementary figures and images for: Influence of age, time of day, and environmental changes on vocalization patterns in broiler chickens
Source: Poult Sci. 2025 May 14;104(8):105298. doi: 10.1016/j.psj.2025.105298 (PMC12173062; doi:10.1016/j.psj.2025.105298)

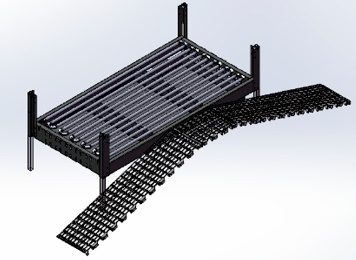

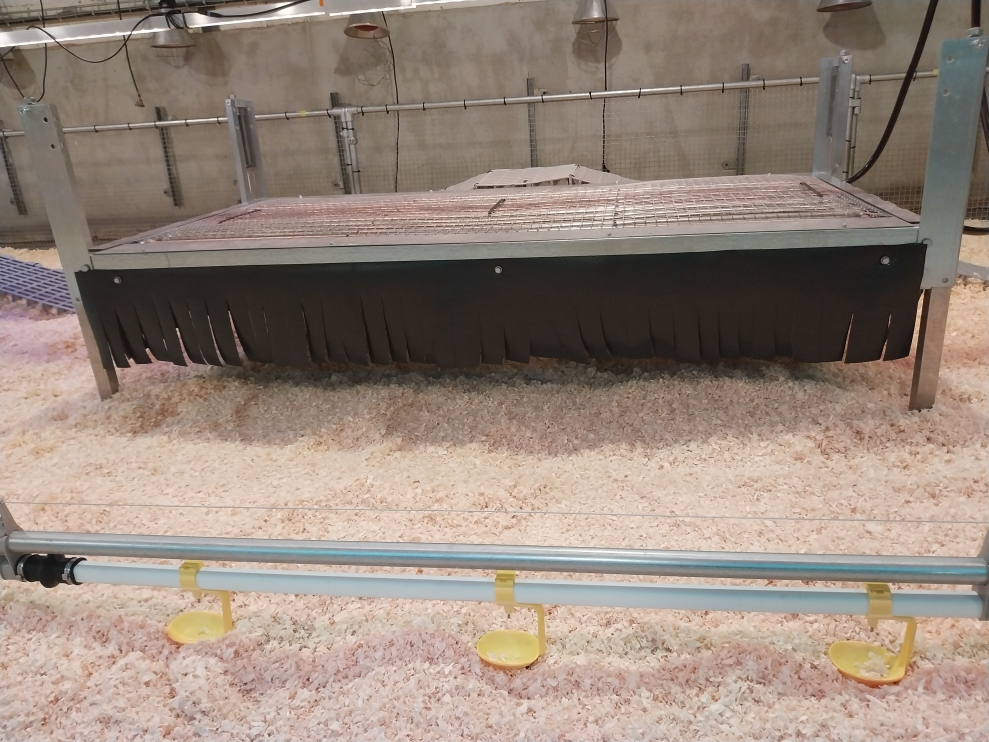

Supplement: Supplementary file 2 [file mmc2.docx]

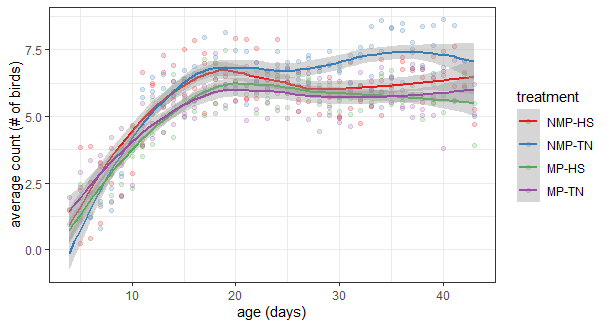

Supplement: Supplementary file 3 [file mmc3.docx]
